# Safety of combined PD‐1 pathway inhibition and radiation therapy for non‐small‐cell lung cancer: A multicentric retrospective study from the GFPC

**DOI:** 10.1002/cam4.1825

**Published:** 2018-10-11

**Authors:** Paul Lesueur, Alexandre Escande, Juliette Thariat, Enora Vauléon, Isabelle Monnet, Alexis Cortot, Delphine Lerouge, Serge Danhier, Pascal Dô, Catherine Dubos‐Arvis, Christos Chouaïd, Radj Gervais

**Affiliations:** ^1^ Radiation Oncology Centre Francois Baclesse Centre de Lutte Contre le Cancer Caen France; ^2^ Radiation Oncology Centre Oscar Lambret Lille France; ^3^ Medical Oncology Centre Oscar Lambret Lille France; ^4^ Onco‐pneumology Centre Hospitalier Intercommunal de Creteil Île‐de‐France France; ^5^ Pneumology Centre Hospitalier Regional Universitaire de Lille Lille France; ^6^ Onco‐pneumology Centre Francois Baclesse Centre de Lutte Contre le Cancer Caen France

**Keywords:** anti‐PD‐1, checkpoint inhibitor: radiotherapy, combination, nivolumab, safety

## Abstract

**Introduction:**

Randomized prospective studies on patients with metastatic non‐small‐cell lung cancers (NSCLCs) showed that anti‐programmed death‐1 (PD‐1) agents notably improved 2‐year overall survival (OS) rates, compared to docetaxel. NSCLC patients now receive nivolumab and irradiation, concurrently or not. However, little is known about the safety of this combination, even though the preclinical model suggested a possible synergic effect. We analyzed NSCLC patients treated with radiotherapy and nivolumab according to former's timing.

**Methods:**

We retrospectively reviewed records of a large series of metastatic NSCLC patients from three French centers, irradiated during the 6 months preceding, concomitantly, or 3 months after nivolumab administration to assess nivolumab tolerance and outcomes.

**Results:**

Among 104 patients included (37 women; 67 men; median age 60.3 years; 67% with performance status <2; 93.2% were current or past smokers) and their 144 intra‐ or extracranial irradiation courses, any‐grade adverse events (AEs) were observed in 62 (59.6%), with 10 (9.6%) experiencing at least one grade 3/4 toxicity and 9 (8.7%) at least one grade 3/4 immune‐related AE (IRAE). Respective 1‐ and 2‐year OS rates were 48.8% and 29.1%, while 1‐ and 2‐year progression‐free survival (PFS) rates were 20.9% and 10.1%. PFS was significantly better for patients with IRAE(s) (*P* = 0.038) than those without and a trend toward better OS (*P* = 0.06). Delivering radiation before or during/after nivolumab administration was not associated with better OS or PFS.

**Conclusion:**

Radiotherapy delivered during the 6 months before, during, or the three months following nivolumab for NSCLCs was not associated with an increased risk of severe or unexpected toxicities.

## 1 INTRODUCTION

Immune checkpoint inhibitors are considered a major advance in the treatment of various cancers with poor prognoses. Anti‐cytotoxic T‐lymphocyte protein‐4 (CTLA‐4) and anti‐programed death‐1 (PD‐1)/PD‐ligand‐1(PD‐L1) are the two most developed families of those inhibitors. They act by stimulating the host immune system to eliminate tumor cells through recruitment and activation of cytotoxic effector cells, thereby preventing CTLA‐4‐CD80/CD86 and PD‐1‐PD‐L1 interactions.^1^, ^2^

Anti‐PD‐1 nivolumab has demonstrated improved overall survival (OS) among patients with advanced non‐small‐cell lung cancers (NSCLCs). Indeed, CheckMate017 and CheckMate057 trials showed notably improved 2‐year OS rates.^3^, ^4^ Second‐line nivolumab vs docetaxel had 2‐year OS rates of 23% vs 8% for patients with squamous NSCLCs and 29% vs 16% for those within non‐squamous NSCLCs.^5^ Consequently, nivolumab is now considered the new standard of care for second‐line NSCLC treatment.

For patients with advanced NSCLCs, irradiation is routinely prescribed as curative or palliative treatment. At present, many patients receive nivolumab and irradiation, concomitantly or not, during the course of their disease, even though few data are available about this combination's tolerance.

The irradiation dose required to achieve complete tumor regression is often less than the dose expected to kill all the cancer cells, suggesting that irradiation also activates tumoricidal mechanisms other than simply damaging DNA. Irradiation induces innate and adaptive immune responses against antigenic cancer cells that have immunosuppressive mechanisms to escape destruction. According to preclinical models, it also activates inflammatory pathways, facilitates dendritic cell maturation, increases T‐cell priming, and sensitizes tumor cells to immune recognition.^6^, ^7^ To augment irradiation‐induced antitumor immune responses, preclinical and clinical studies focused on combining checkpoint agonists or antagonists with irradiation.^10^ In vivo study results showed that PD‐1 inhibition combined with irradiation led to fewer tumor‐infiltrating myeloid‐derived suppressor cells in the lesion's microenvironment and that anti‐PD‐1 was able to block local suppression of the irradiation‐induced immune response,^11^, ^12^ leading to higher tumor response rates. Outcomes of some retrospective studies using that combination with intra‐ or extracranial palliative irradiation, mostly to treat melanomas, indicated no excessive anti‐PD‐1 or radiotherapy toxicity.^13^, ^15^, ^16^ Clinical trials testing the combination of irradiation and PD‐1/PD‐L1 inhibition have been initiated, but the results are not yet available.

Pending the outcomes of prospective trials, we undertook this study to evaluate the combination of radiotherapy and nivolumab tolerance and outcomes on a large series of NSCLC patients treated according to routine practice.

## 2 MATERIALS AND METHODS

### 2.1 Population and inclusion criteria

Medical charts of consecutive NSCLC patients who had received nivolumab since 2014 in three French institutions were retrospectively screened for eligibility. The main inclusion criteria for patients were as follows: >18 years old, with histologically proven NSCLC, who had received at least one nivolumab infusion and irradiation, 6 months before, or concomitantly (meaning during or between nivolumab perfusions) or 3 months after the last nivolumab infusion (henceforth during/after). The two groups, before and during/after, were compared. The 6 months before nivolumab take account of irradiation‐induced systemic immune cell modification^18^ and the 3 months post‐nivolumab, assure the complete elimination of nivolumab (five half‐lives).^19^ No restrictions addressed the irradiated target or irradiation modality: hypofractionated stereotactic radiotherapy (HFSR), stereotactic radiosurgery (SRS), intensity‐modulated radiotherapy (IMRT), or three‐dimensional conformational radiotherapy (3DCRT) could be used. The number of irradiation cycles per patient and their indication is reported. Irradiation cycle preparation and dosimetry adhered to local practices. Follow‐up was calculated from the first nivolumab infusion. To examine potential relationships between irradiation timing and nivolumab tolerance and efficacy, we compared overall survival (OS) and progression‐free survival (PFS) outcomes of patients who had received irradiation before vs during/after receiving the immune checkpoint inhibitor. Patients were followed by local medical and/or radiation oncologist(s). Adverse events (AEs) were assessed according to CTCAE 4.0 criteria. Immune‐related AEs (IRAEs) were managed according to recommended algorithms,^20^ usually with high‐dose corticosteroids and prolonged tapering for patients who developed more severe AEs.

Local control was defined according to RECIST 1.1 criteria. For bone lesions, local control was defined as an improvement of local pain, and the absence of skeletal‐related events or unequivocal radiological progression, after irradiation. This study was approved by French Ethics Committees and the National Commission on informatics and Liberties (MR003 Methodology).

### 2.2 Statistical analyses

Descriptive parameters are expressed as number (%) and median (interquartile 25th‐75th percentiles), unless stated otherwise. Kaplan‐Meier OS estimates were calculated from the first metastasis diagnosis and from the first nivolumab infusion. Kaplan‐Meier PFS and in‐field PFS (IF‐PFS) estimates were calculated, respectively, from the first nivolumab infusion and from the end of external beam radiotherapy (EBRT). Log‐rank tests or Cox regression models were used to test the impact of factors on OS. Hazard assumptions were validated before analysis. Only nonassociated factors with *P* < 0.1 were included in multivariate analyses. Restricted mean survival time (RMST) tests, used to evaluate the impact of the therapeutic sequence on OS and PFS, are an alternative robust and clinically interpretable summary measure that does not rely on the proportional hazard assumption.^21^, ^22^ Associations between characteristics and toxicity were calculated using Spearman's nullity, Wilcoxon‐Mann‐Whitney, and chi‐square tests, depending on the type of factor, with *P* < 0.05 defining statistical significance. Statistical analyses were computed using R studio, version 3.3.3 (R Studio Team (2016). R Studio: Integrated Development for R. R Studio Inc., Boston, MA).

## 3 RESULTS

### 3.1 Patient characteristics

One hundred and four NSCLC patients were included (64.4% male; median age: 60.3 years; 67% with performance status (PS) <2, 93.2% current or past smokers) (Table 1). At nivolumab onset, 46 (44.2%) had brain metastases and 24 (23.1%) had >2 metastatic sites.

**Table 1:** Characteristics of 104 NSCLC patients treated with Anti‐PD‐1 and radiotherapy

Characteristic	Value
Age at diagnosis, y	60.3 (54.5‐67.1)
Sex	
Female	37 (35.6%)
Male	67 (64.4%)
Current or past smoker	96 (92.3%)
Age at distant disease diagnosis, y	60.9 (54.5‐68.3)
Performance status at nivolumab onset	
0	16 (15.4%)
1	53 (51%)
2‐3	35 (33.5%)
Tumor	
Histological type	
Squamous cell carcinoma	65 (62.5%)
Adenocarcinoma	34 (32.7%)
Others	5 (4.8%)
Mutation	34 (32.7%)
*KRAS*	22 (21.2%)
*EGFR*	2 (1.9%)
*ALK*	2 (1.9%)
*MET*	5 (4.8%)
Others (*BRAF*,* HER2*…)	3 (2.9%)
Brain metastasis	46 (44.2%)
Number of different disease sites	2 (2‐2)
1	21 (20.2%)
2	55 (52.9%)
≥3	24 (23.1%)

### 3.2 Treatment characteristics

The majority of patients (54.4%) received nivolumab as second‐line treatment for metastatic NSCLC; a median of 5 (3‐11) infusions was administered. The main reason for stopping nivolumab was disease progression (52.9%). Fifty‐nine patients received irradiation during the 6 months before nivolumab and 45 underwent irradiation during/after nivolumab treatment. It is worth to note that these two groups were statistically comparable. The 104 patients received 144 irradiation cycles (median one cycle/patient delivered to a median of one target lesion/patient). Bone (48.6%) and brain (31.3%) were the two most irradiated sites. 3DCRT was the most used technique, for 75% of the courses. Stereotactic radiotherapy was delivered to 28 targets with different administration schedules (3 × 10 Gy, 1 × 20‐25, 6 × 6…). A median dose of 30 Gy was delivered in 10 fractions. Radiotherapy with palliative intent for pain or compression was delivered in 108 of 144 (75%) cycles. The two most used schedules were, then, 10 × 3Gy (n = 65) and 5 × 4Gy (n = 16) Twenty‐six of 144 (18%) target lesions were irradiated for asymptomatic oligo‐progressive or oligo‐metastatic disease, and 8 of 144 (5.6%) irradiation treatments were delivered in the context of local‐regional curative chemo‐radiotherapy or post‐surgery thoracic adjuvant radiotherapy (Table 2). Two patients (1.4%) received a high‐dose (60‐66 Gy) thoracic irradiation after having obtained with nivolumab an excellent partial response and an extended stable disease Thirty‐five of 144 (24.3%) irradiation cycles were delivered during nivolumab treatment. Fourteen patients were still taking nivolumab at the time of this analysis.

**Table 2:** Characteristics of the anti‐PD‐1 and irradiation treatments given to 104 patients

Treatment characteristic	Value*^a^*
Systemic	
Number lines before nivolumab	
0‐1	57 (54.8%)
2	31 (29.8%)
≥3	16 (15.4%)
Number of nivolumab cycles	5 (3‐11)
EBRT (/144) between during nivolumab administration	35 (25.4%)
Causes of nivolumab stoppage	
Death	9 (8.7%)
Performance status	9 (8.7%)
Progression	55 (52.9%)
Temporarily suspended	4 (3.8%)
Toxicity	11 (10.6%)
Other	2 (1.9%)
EBRT	
Prior EBRT (>6 mo before nivolumab)	79 (76%)
Total number of irradiation cycles per patient	1 (1‐1)
1	88 (84.6%)
2	11 (10.6%)
3	4 (3.8%)
4	1 (1.0%)
Total number of irradiated targets per patient	1 (1‐1)
1	79 (76%)
2	17 (16.3%)
≥3	8 (7.7%)
Curative intend EBRT/144 cycles	8 (5.6/%)
Symptomatic palliative EBRT/144 cycles	108 (75%)
Asymptomatic palliative EBRT /144 cycles	26 (18%)
Closing EBRT /144 cycles	2 (1.4%)
Timing of irradiation	
Before nivolumab	59 (56.7%)
During/after nivolumab	45 (43.3%)
Radiotherapy technique per cycles	
3DCRT	109 (75.7%)/144
SRS	28 (19.4%)
IMRT	6 (4.2%)
Other	1 (0.7%)
Dose (Gy)	30 (29.6‐30.0)
BED(Gy)	39 (39‐51)
EQD2 (Gy)	33 (33‐43)
Number of fractions	10 (5‐10)
Irradiated sites	144
Bone	70 (48.6)
Brain	45 (31.3%)
Lung	18 (12.5%)
Others	11 (7.6%)

### 3.3 Treatment toxicity

Sixty‐two (59.6%) patients reported at least one AE and 10 at least one grade 3/4 AE. Forty‐seven (45.2%) patients reported at least one IRAE without any grade 5 toxicity. The only grade 3 radiation‐related EA occurred after a whole‐brain radiotherapy and consisted in an intracranial hypertension. Ten (9.7%) patients stopped anti‐PD‐1 therapy because of toxicity. Univariate analysis, looking for factor(s) predictive of toxicity, showed that age, sex, number of nivolumab infusions, number of previous lines, and PS were not associated with a higher toxicity rate. Patients receiving irradiation during the 6 months before the first nivolumab infusion presented the same grade 3/4 AE risk as those who received radiotherapy during/after nivolumab (*P* = 0.51). Only one grade 3 IRAE, that is, immune esophagitis, corresponded to the irradiation field (Table 3).

**Table 3:** NSCLC patients’ tolerance of Nivolumab and irradiation

AEs (n = 90)	Grade 1/2	Grade 3/4
All		
Overall	77 (85.6%)	13 (14.4%)
Pulmonary	3 (3.3%)	1 (1.1%)
Gastrointestinal	20 (22.2%)	1 (1.1%)
Dermatological	11 (12.2%)	2 (2.2%)
Endocrinological	8 (8.9%)	2 (2.2%)
Rheumatological	5 (5.6%)	1 (1.1%)
Asthenia	16 (17.8%)	3 (3.3%)
Hematological	0 (0%)	1 (1.1%)
Others	14 (15.6%)	2 (2.2%)
Nivolumab‐induced (n = 65)		
Overall	53 (81.5%)	12 (18.5%)
Pulmonary	3 (4.6%)	1 (1.5%)
Gastrointestinal	8 (12.3%)	1 (1.5%)
Dermatological	4 (6.2%)	2 (3.1%)
Endocrinological	8 (12.3%)	2 (3.1%)
Rheumatological	5 (7.7%)	1 (1.5%)
Asthenia	14 (21.5%)	3 (4.6%)
Hematological	0 (0%)	1 (1.5%)
Others	11 (16.9%)	1 (1.5%)

### 3.4 OS and PFS

**Figure 1:**
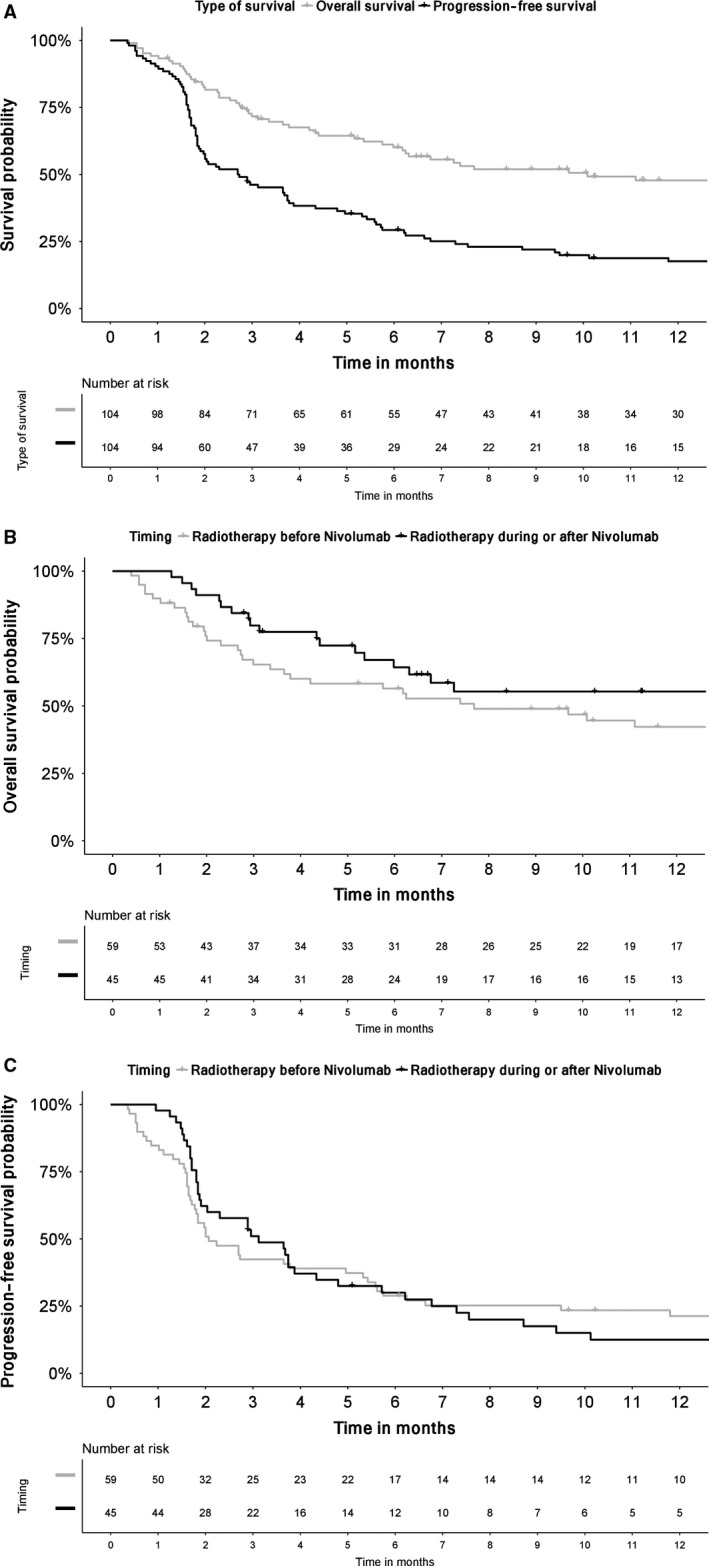
Estimated Kaplan‐Meier overall survival (OS) and progression‐free survival (PFS) probabilities (%) (A) in years, and (B) OS and (C) PFS probabilities (depending on EBRT timing in relationship to nivolumab onset) in months since starting anti‐PD‐1

At median follow‐up of 15.8 (95% confidence interval (CI): 12.24‐19.4) months, median OS was 11.1 (95% CI: 5.8‐16.5) months since the first nivolumab infusion and 2.1 (95% CI: 1.6‐2.7) years since metastasis diagnosis, median PFS was 2.7 (95% CI: 1.4‐4.1) months, and respective 1‐ and 2‐year OS rates were 47.8% and 29.5%, and PFS rates were 17.6% and 10.2% (Figure 1A).

At the time of analysis, 144 irradiated targets were analyzed, 28 (19.4%) in‐field relapses had occurred, with 64.4% 1‐ and 2‐year local control rates of irradiated sites (Table 4). According to univariate analyses, OS and PFS did not seem to be associated with the timing of irradiation delivery. Indeed, 1‐year OS for patients who had received radiotherapy during the 6 months before nivolumab was 55.3% vs 42.2% when irradiation was delivered during/after nivolumab (*P* = 0.39), with respective 1‐year PFS rates of 21.3% and 12.5% (*P* = 0.90). Among the other potential prognostic factors tested in univariate analyses (sex, PS, histology, tobacco, number of metastatic sites, the presence of brain metastases), only PS <2 at nivolumab onset was predictive of longer PFS (*P* = 0.047). PFS was significantly better for patients with IRAE(s) (*P* = 0.038) than those without and a trend toward better OS (*P* = 0.06).

**Table 4:** Outcomes for the 104 NSCLC patients

Parameter	1‐y survival (95% CI)	2‐y survival (95% CI)
OS since metastasis diagnosis	79.5% (71.7‐87.3)	45.9% (35.1‐56.7)
OS since starting nivolumab	47.8% (38.5‐59.3)	29.5% (18.9‐45.9)
PFS since starting nivolumab	17.6% (11.5‐27.0)	10.2% (5.3‐19.7)
Local control rate, %	64.4% (52.2‐76.6)	64.4% (52.2‐76.6)
In‐field PFS, %	34.8% (24.8‐44.8)	17.9% (4.2‐31.8)
EBRT before nivolumab		
OS	55.3% (41.6‐73.6)†	34.0% (20.3‐57.0)
PFS	21.3% (12.9‐35.1)‡	12.8% (5.8‐28)
EBRT during/after nivolumab		
OS	42.2% (30.8‐57.9)†	27.2% (14.4‐51.1)
PFS	12.5% (5.6‐28)‡	6.6 (2‐22.5)

a Cox regression model: *P* = 0.390.

b Cox regression model: *P *= 0.900.

Results of multivariate analyses are reported in Table 5. Given that the proportional hazard assumption was not respected for PFS, RMST tests at 12 months were computed and showed that sequence timing had no impact on OS (*P* = 0.180) or PFS (*P* = 0.923).

**Table 5:** Univariate and multivariate analyses of survival‐associated parameters since nivolumab onset for the 104 NSCLC patients

Parameter	OS HR (95% CI)	*P* value	PFS HR (95% CI)	*P* value
Univariate				
Sex	0.85 (0.55‐0.62)	0.845	0.91 (0.59‐1.41)	0.685
Smoker	1.07 (0.39‐2.96)	0.895	0.95 (0.44‐2.06)	0.898
Histology	0.78 (0.48‐1.24)	0.565	0.80 (0.55‐1.14)	0.459
Performance status >1	2.04 (0.87‐4.75)	0.091	1.88 (0.99‐3.53)	0.047
Brain metastasis	0.96 (0.57‐1.63)	0.906	1.22 (0.80‐1.86)	0.298
Anti‐PD‐1 adverse event	0.61 (0.36‐1.03)	0.064	0.64 (0.42‐0.98)	0.038
Multivariate				
Performance status >1	1.913 (0.818‐4.475)	0.13	1.81 (0.960‐3.418)	0.07
Anti‐PD‐1 adverse event	0.640 (0.377‐1.087)	0.09	0.66 (0.433‐1.099)	0.06

HR, hazard ratio.

### 3.5 Impact of the irradiation site

The delivery of extracranial radiotherapy, in comparison with brain radiotherapy, did not impact the patient's outcomes (1‐year OS 44.2% vs 33.3%, *P* = 0.71; and 1‐year PFS 21.4% vs 16.2%, *P* = 0.54). Similarly, considering OS and PFS, there was no difference between patients who received at least one bone irradiation vs those who never received any bone irradiation. (*P* = 0.58 for OS and *P* = 0.13 for PFS).

## 4 DISCUSSION

Although used in routine practice, the tolerance of radiotherapy and anti‐PD‐1 therapy, particularly nivolumab, for patients with metastatic or locally advanced NSCLCs is not well‐known. The analysis of 104 patients, who had received 144 radiotherapy courses 6 months before, during/after the 3 months following nivolumab, did not reveal any particular irradiation timing‐associated toxicity, with only 9.6% of the patients developing grade 3/4 AEs. For our series, the any‐grade IRAE rate was 45.2%, in line with pooled CheckMate‐017 and CheckMate‐057 analysis (68%; with 10% grade 3/4).^5^ With 2‐year OS at 29.1%, the outcomes of this real‐life series were also in accordance with pivotal study results, despite our patients having been more intensively treated than in the clinical trials (55.2% received nivolumab beyond the second line and 44.2% had brain metastases). This good tolerance of the combination of radiotherapy and anti‐PD‐1 has been found previously.^13^, ^15^, ^16^, ^23^, ^24^ In a retrospective analysis of 163 patients with advanced NSCLCs and brain metastases, rates of all‐grade AEs and grade ≥3 AEs did not differ significantly between patients, who received intracranial RT and were treated with or without anti‐PD‐1 (grade ≥3 AEs: 8% of anti‐PD‐1‐naïve patients vs 9% of anti‐PD‐1‐treated patients with SRS, *P* = 1.00; and 8% of anti‐PD‐1‐naïve patients vs 0% of anti‐PD‐1‐treated patients with whole‐brain RT, *P* = 0.71). In addition, AE rates did not differ according to the timing of anti‐PD‐1 administration with respect to irradiation.^23^ Our results also agreed with the Keynote‐001 trial subset analysis of irradiated patients^26^ and other retrospective series.^24^, ^27^ Only one patient experimented a grade III immune‐related adverse event directly correlated to the irradiation field (immune esophagitis after a cervical irradiation). This kind of secondary effect could be related to a T‐cell infiltration of the esophagus according to the hypothesis proposed by Myers and Du, after having exposed murine preclinical model to the therapeutic combination.^28^, ^29^ In their studies, T‐cell counts were significantly elevated in both cardiac and pulmonary tissues after combination therapy as compared to treatment with radiation alone, indicating that, while prolonging the action of immune cells may enhance their antitumor activity, nonmalignant tissue damaged by irradiation is susceptible to accumulation of and further damage by activated T cells. It could be one of the limits of this treatment combination in clinical practice. Indeed, in the PACIFIC study, the most frequent adverse events leading to discontinuation of durvalumab and placebo were pneumonitis or radiation pneumonitis (in 6.3% and 4.3%, respectively). For patients who received durvalumab after concomitant chemo‐radiotherapy, in comparison with those who received placebo, radiation‐induced pneumonitis or pneumonitis occured more frequently (33.9% vs 24.8%).^30^

Recently, Bang et al^17^ analyzed a heterogeneous mix of 133 patients, 71 with NSCLCs, treated with radiation and immune checkpoint blockade (anti‐PD‐1, anti‐PD‐L1, or anti‐CTLA‐4). For patients receiving anti‐PD‐1 and radiotherapy, the grade 3/4 toxicity rate was 4%, a little bit lower than ours, but they did not apply time restrictive inclusion criteria. Similarly, our patients given radiotherapy before nivolumab had the same grade 3/4 IRAE risk as patients irradiated during/after nivolumab. According to our analyses, IRAEs were associated with better PFS (*P* = 0.038) and a trend toward longer OS (*P* = 0.06), in agreement with those of Hwang et al, who also found that grade >2 IRAEs in NSCLC patients treated with thoracic radiotherapy and anti‐PD‐1 were associated with longer survival.^24^ That relationship was also described in NSCLC^31^ and melanoma patients.^32^

The synergy of radiotherapy with anti‐PD‐L1 efficacy remains controversial. Among 97 patients with advanced NSCLCs treated in the phase 1 Keynote‐001 trial at the University of California,^27^ 42 (43%) had received extracranial (39%) and thoracic (25%) irradiation before pembrolizumab. With median follow‐up at 32.5 months, PFS with pembrolizumab was significantly longer for patients previously irradiated than those without prior radiotherapy (4.4 vs 2.1 months; *P* = 0.019) and for patients who had previously received extracranial irradiation compared to those without (6.3 vs 2.0 months; *P* = 0.0084). OS with pembrolizumab also was significantly longer for patients with prior radiotherapy than those without (10.7 vs 5.3 months; *P* = 0.026); and for patients previously given extracranial radiotherapy than those without (11.6 vs 5.3 months; *P* = 0.034). No excess toxicity occurred in patients who had received the previous radiotherapy. In the Keynote‐001 subgroup analysis, extracranial irradiation led to higher OS improvement, than cerebral irradiation. In our study, there was no impact of the irradiation site. In a multicenter, retrospective, cohort study, analyzing 146 consecutive patients treated with nivolumab, 56 with prior radiotherapy; no PFS difference was found between patients with and without prior radiotherapy.^33^

Although preclinical models indicate that combining irradiation with anti‐PD‐L1 concurrently or at least very close to immune checkpoint inhibitor administration is optimal; however, the question persists in clinical practice.^34^

According to our restricted mean survival analysis, the therapeutic sequence (radiotherapy before nivolumab vs. radiotherapy during/after nivolumab) did not impact outcomes. Similar conclusions were drawn based on a cohort of 53 melanoma patients treated with radiotherapy and anti‐PD‐1 (nivolumab or pembrolizumab).^15^ Neither response rates nor OS differed between patients given concurrent or sequential radiotherapy. However, compared to the sequential combination, Qin et al described longer irradiated tumor responses when radiotherapy was delivered after ipilimumab, whereas for Kiess et al, concurrently delivered ipilimumab and SRS was associated with favorable local‐regional control and perhaps OS.^35^, ^36^ It is worth noting that those studies did not apply restrictive inclusion criteria concerning the interval between checkpoint inhibitor administration and radiotherapy and, for some patients, it exceeded 3 years, which renders interpretation of the definitive results more difficult.

Like any other retrospective study, there are some limits with our study. Patients received fewer nivolumab infusion (5 cycles, range, 1‐50) in comparison with checkmate studies. In fact, in the pivotal trial, the median administered cycles were 6 doses (range, 1‐52) for non‐squamous NSCLC and 8 doses (range, 1‐48) for squamous NSCLC. However, in our study, the studied population is a real‐life cohort with PS2 patients, patients with cerebral metastasis and more comorbidities compared to patients included in phase III pivotal trials. It could explain the difference observed and maybe minimize the toxicity rates.

Unfortunately, we were not able to define an optimal dose or fractionation to improve patients’ outcomes and provide the best systemic immunomodulation. Indications of radiotherapy were too heterogeneous (even among palliative intent irradiations), to make robust conclusions.

Additional clinical trials investigating the combination of radiotherapy and immune checkpoint inhibitors are needed to determine the optimal therapeutic strategy for patients with advanced NSCLCs.

## 5 CONCLUSION

To conclude, the combination of nivolumab with radiotherapy for NSCLC patients was not associated with a heightened risk of severe or unexpected AEs, attributable to nivolumab, or any irradiation modality. Pending results from prospective or randomized controlled trials, our results can reassure physicians about prescribing this combination in routine practice, when palliative irradiation is necessary.

## CONFLICT OF INTERESTS

None declared.
