# Corrigendum

**DOI:** 10.1002/cam4.2427

**Published:** 2019-08-06

In the article by Lesueur et al,^1^ the authors regret that the published version of Table 4 and the text in the second paragraph of section “3.4 OS and PFS” were incorrect. A mismatch in the text and data in the table has been noticed.

The correct text in section 3.4 and data in Table 4 are shown below:

## 3.4 | OS and PFS

At median follow‐up of 15.8 (95% confidence interval (CI): 12.24‐19.4) months, median OS was 11.1 (95% CI: 5.8‐16.5) months since the first nivolumab infusion and 2.1 (95% CI: 1.6‐2.7) years since metastasis diagnosis, median PFS was 2.7 (95% CI: 1.4‐4.1) months, and respective 1‐ and 2‐year OS rates were 47.8% and 29.5%, and PFS rates were 17.6% and 10.2% (Figure 1A).

At the time of analysis, 144 irradiated targets were analyzed, 28 (19.4%) in‐field relapses had occurred, with 64.4% 1‐ and 2‐year local control rates of irradiated sites (Table 4). According to univariate analyses, OS and PFS did not seem to be associated with the timing of irradiation delivery. Indeed, 1‐year OS for patients who had received radiotherapy during the 6 months before nivolumab was 42.2% vs 55.3% when irradiation was delivered during/after nivolumab (*P* = 0.39), with respective 1‐year PFS rates of 21.3% and 12.5% (*P* = 0.90). Among the other potential prognostic factors tested in univariate analyses (sex, PS, histology, tobacco, number of metastatic sites, the presence of brain metastases), only PS <2 at nivolumab onset was predictive of longer PFS (*P* = 0.047). PFS was significantly better for patients with IRAE(s) (*P* = 0.038) than those without and a trend toward better OS (*P* = 0.06).

Results of multivariate analyses are reported in Table 5. Given that the proportional hazard assumption was not respected for PFS, RMST tests at 12 months were computed and showed that sequence timing had no impact on OS (*P* = 0.180) or PFS (*P* = 0.923).

**Table 4:** Outcomes for the 104 NSCLC patients

Parameter	1‐y survival (95% CI)	2‐y survival (95% CI)
OS since metastasis diagnosis	79.5% (71.7‐87.3)	45.9% (35.1‐56.7)
OS since starting nivolumab	47.8% (38.5‐59.3)	29.5% (18.9‐45.9)
PFS since starting nivolumab	17.6% (11.5‐27.0)	10.2% (5.3‐19.7)
Local control rate, %	64.4% (52.2‐76.6)	64.4% (52.2‐76.6)
In‐field PFS, %	34.8% (24.8‐44.8)	17.9% (4.2‐31.8)
EBRT before nivolumab		
OS	42.2% (30.8‐57.9)†	27.2% (14.4‐51.1)
PFS	21.3% (12.9‐35.1)‡	12.8% (5.8‐28)
EBRT during/after nivolumab		
OS	55.3% (41.6‐73.6) †	34.0% (20.3‐57.0)
PFS	12.5% (5.6‐28)‡	6.6 (2‐22.5)

^†^ Cox regression model: *P* = 0.390.

^‡^ Cox regression model: *P* = 0.900.

The authors would like to apologize for any inconvenience that it had caused.
